# Dysbiosis associated with acute helminth infections in herbivorous youngstock – observations and implications

**DOI:** 10.1038/s41598-019-47204-6

**Published:** 2019-07-31

**Authors:** Laura E. Peachey, Cecilia Castro, Rebecca A. Molena, Timothy P. Jenkins, Julian L. Griffin, Cinzia Cantacessi

**Affiliations:** 1 Department of Veterinary Medicine, University of Cambridge, Cambridge, United Kingdom; 2 Bristol Veterinary School, University of Bristol, Langford, United Kingdom; 3 Department of Biochemistry, University of Cambridge, Cambridge, United Kingdom

**Keywords:** Bacteriology, Parasite host response

## Abstract

A plethora of data points towards a role of the gastrointestinal (GI) microbiota of neonatal and young vertebrates in supporting the development and regulation of the host immune system. However, knowledge of the impact that infections by GI helminths exert on the developing microbiota of juvenile hosts is, thus far, limited. This study investigates, for the first time, the associations between acute infections by GI helminths and the faecal microbial and metabolic profiles of a cohort of equine youngstock, prior to and following treatment with parasiticides (ivermectin). We observed that high *versus* low parasite burdens (measured *via* parasite egg counts in faecal samples) were associated with specific compositional alterations of the developing microbiome; in particular, the faecal microbiota of animals with heavy worm infection burdens was characterised by lower microbial richness, and alterations to the relative abundances of bacterial taxa with immune-modulatory functions. Amino acids and glucose were increased in faecal samples from the same cohort, which indicated the likely occurrence of intestinal malabsorption. These data support the hypothesis that GI helminth infections in young livestock are associated with significant alterations to the GI microbiota, which may impact on both metabolism and development of acquired immunity. This knowledge will direct future studies aimed to identify the long-term impact of infection-induced alterations of the GI microbiota in young livestock.

## Introduction

A wealth of data supports the primary role(s) that the vertebrate gastrointestinal (GI) microbiome plays in overall host health^1–3^. In humans, microbial populations inhabiting the gut are acquired during early life, and a ‘stable’ gut microbiome is established by 31 months of age^4,5^. A similar process has been reported in other animals, with the timing of maturation and development of the gut microbiome depending on species lifespan and average age at weaning^6,7^. A balanced gut microbiota is key to vertebrate long-term health and wellbeing; nevertheless, several factors and life events may impact on the establishment of a ‘healthy’ gut flora in early life. Such factors include, but are not limited to, early life nutrition, use of antimicrobials during gestation and/or in young animals, mode of delivery and infectious diseases^8–23^.

In neonates and young animals, perturbations of the developing GI microbiota are known to pre-dispose to the onset of a number of systemic conditions, such as allergies, autoimmunity, obesity and colonisation by infectious agents, such as parasites^24–29^. The intimate mechanisms governing the relationships between host, gut microbiota and parasites are complex and, in many cases, not fully understood; however, experimental evidence points towards a role of the microbiota in supporting the development and regulation of the host immature immune system, e.g. ensuring that adequate responses are mounted against pathogenic stimuli^1,30^. Indeed, recent data generated from an amphibian model of parasite infection demonstrated that susceptibility to colonisation by the helminth *Aplectana hamatospicula* of adult animals was directly attributable to a reduced diversity of the microbial populations inhabiting the gut of juveniles^27^. On the other hand, helminth parasites have been shown to interact, directly and/or indirectly, with the gut flora of their vertebrate hosts^31–42^, and thus could potentially interfere with the establishment of a ‘healthy’ microbiome in young humans and animals from endemic areas. For instance, experimental infections of rodents with the whipworm *Trichuris muris* (a model for human infections caused by *T*. *trichiura*) and the human blood fluke *Schistosoma mansoni* have been associated with drastic reductions in GI microbial diversity^36–43^, with likely negative implications for microbiome maturation in juvenile hosts, and thus for long term immune and metabolic homeostasis. Beside humans, such effects are likely to have particularly severe repercussions in managed livestock herbivore species, e.g. ruminants and equines, that often harbour high burdens of GI helminths due to slow development of acquired immunity and high transmission rates within herds^44,45^. Thus far, very few studies have examined the impact of helminth infections on the GI microbiota of herbivorous livestock, with inconsistent findings^40,46–48^. In particular, infection of calves (3–4 months old (mo)) and goats (3 mo) with the abomasal parasites *Ostertagia ostertagi*^47^ and *Haemonchus contortus*^46^, respectively, showed no effect of the infection on microbial diversity at the site of parasite establishment, whilst a study of *H*. *contortus* infection in lambs (3 mo), reported a transient increase in gut bacterial alpha diversity associated with parasite colonisation^48^. Clearly, further studies are necessary to elucidate the impact that infections with large burdens of parasitic helminths exert on the developing microbiome of juvenile vertebrates and, in turn, on host long-term health and wellbeing.

Managed equine youngstock provide an ideal system for such investigations; indeed, domestic horses are characterised by long lifespans when compared to other livestock species, thus enabling comparisons between the microbiota of young *versus* adult stock within herds and long-term monitoring of health parameters. In addition, equines are often subjected to strict diets, which provides an opportunity to overcome confounding factors linked to diet variability. Finally, the equine GI microbiome has been extensively characterised in both health and disease^7,49–69^, thus providing a benchmark for comparative analyses with microbiome composition in parasite-infected horses.

Therefore, in order to identify changes to GI microbial composition, diversity and function associated with GI helminth infection in young herbivorous livestock, our study characterised, for the first time, the faecal microbiome and metabolome of a cohort of thoroughbred (TB) youngstock acutely infected with an economically important group of GI helminths (i.e. the Cyathostominae, strongyle parasites characterised by a direct, non-migratory life cycle with oro-faecal transmission^70,71^) pre- and post-treatment with anthelmintic compounds.

## Results

### Comparison of faecal microbiota composition in equine youngstock with high *versus* low parasite burdens, prior to and following anthelmintic treatment

A cohort of 53 TB equine youngstock were examined for cyathostomin infection and allocated to high- and low-infection burden groups according to the following criteria: i) faecal parasite egg count (FEC – a proxy of parasite infection burden) of ≥100 eggs per gram (e.p.g.) (=C-high) or ≤10 e.p.g. (C-low) in duplicate faecal samples on day 0 (D0) of the study; (ii) negative for co-infections with other GI helminths; (iii) no antibiotic treatment for at least 2 months prior to sampling. Out of the 53 animals screened, 23 matched these criteria, of which 9 were enrolled into the C-high group and 14 into the C-low (Supplementary Table S1). Samples were collected for analyses of faecal microbiota and metabolites from all animals in the C-high and C-low groups immediately prior to treatment with ivermectin (0.2 mg/kg) at D0, as well as at two (D2) and 14 (D14) days post-treatment. FEC analysis performed on samples collected at D2 and D14 showed FEC reduction rates (FECR) of 100% by D14 in all treated animals (Supplementary Table S1). Following total DNA extraction from faecal samples, bacterial 16S rRNA high-throughput sequencing was performed on each sample. A total of 5,201,731 raw paired-end reads were generated from 74 DNA faecal extracts of C-high and C-low yearlings collected on D0, D2 and D14, respectively, and subjected to further processing. Following primer trimming, joining of paired-end reads, filtering of low-quality sequences and removal of ‘contaminant’ and singleton Operational Taxonomic Units (OTUs), a total of 3,048,051 (mean 43,543; range 21,992–58,947) high-quality sequences were retained for further bioinformatics analyses. The rarefaction curves generated following *in silico* subtraction of low-quality and contaminant sequences indicated that the majority of faecal bacterial communities were represented in the remaining sequence data, thus allowing us to undertake further analyses (Supplementary Fig. S1). These sequences were assigned to 9,972 OTUs and 15 bacterial phyla, respectively. The phyla Bacteroidetes (42.303%) and Firmicutes (42.108%) were predominant in all samples, followed by the phyla Verrucomicrobia (1.319%), Spirochaetes (0.810%), Actinobacteria (0.402%), Proteobacteria (0.3908%), Tenericutes (0.073%), Fibrobacteres (0.0552%), TM7 (0.012%), Lentisphaerae (0.0065%), Synergistetes (0.001%), Fusobacteria (0.001%), Deferribacteres (0.001%), Cyanobacteria (0.001%) and Chlamydiae (0.0001%) (Supplementary Fig. S2), while 11.1% of OTUs could not be assigned to any bacterial group. Predominant sub-taxa were class Bacteroidia, order Bacteroidales within the phylum Bacteroidetes; and class Clostridia, order Clostridiales, families *Lachnospiraceae* and *Ruminococcacae* within the Firmicutes. Given the male-gender bias in the C-high group (Supplementary Table S1), a supervised multivariate Canonical Correlation Analysis (CCA) was performed on the microbial communities detected in samples collected from the whole cohort (C-high and C-low) at D0 with ‘gender’ as an explanatory variable; based on the results of this analysis, gender did not impact significantly on the overall gut microbial composition or alpha diversity of equines enrolled in this study (*P* = 0.826; F = 0.48) (Supplementary Fig. S3).

**Figure 1:**
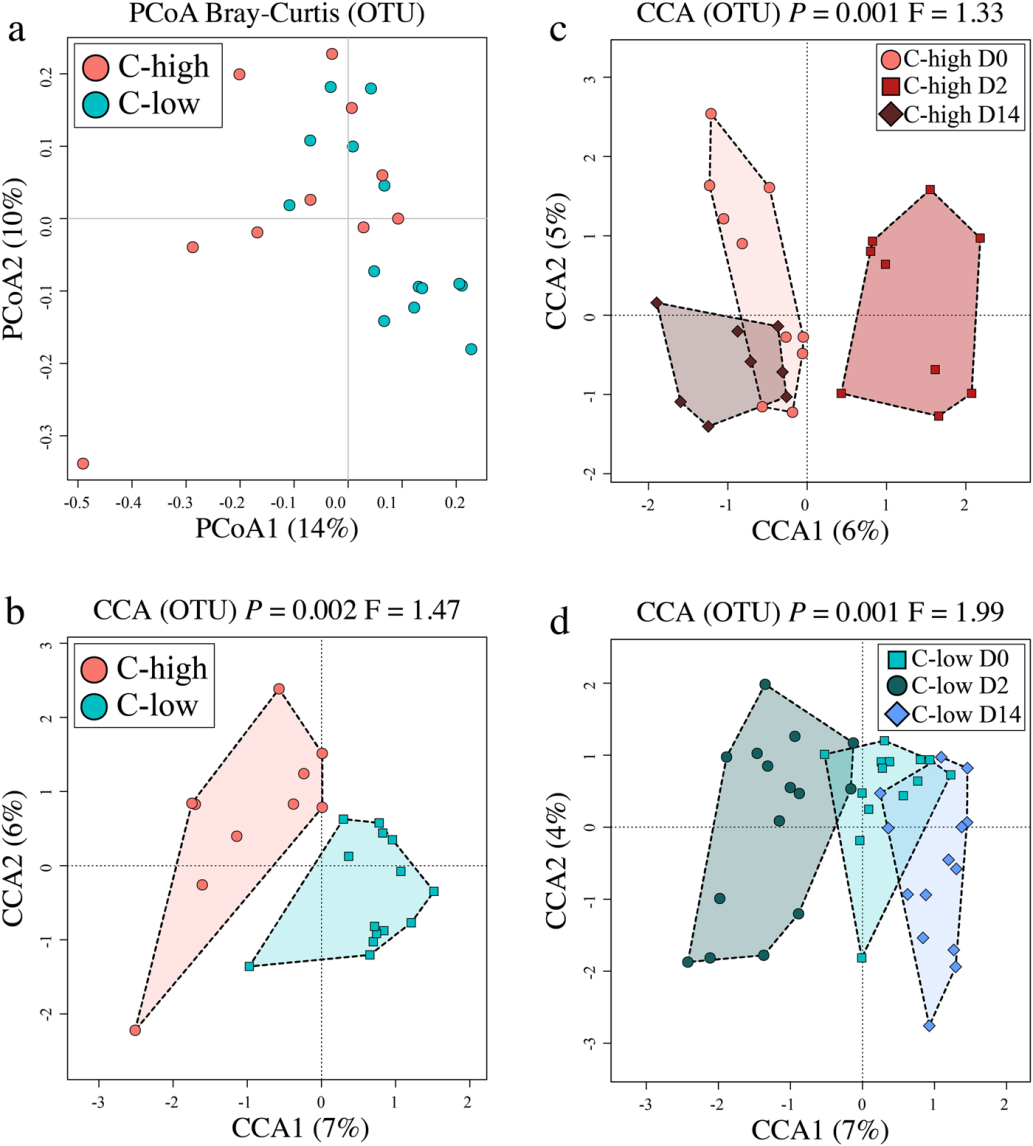
The faecal microbial profiles of youngstock with high (C-high) and low (C-low) parasite infection burdens prior to and following anthelmintic treatment. Multivariate analyses of the faecal microbial composition (based on Operational Taxonomic Unit [OTU] presence and abundance) including (**a**) Principal Coordinates Analyses (PCoA) of faecal microbial profiles from animals in C-high (≥100 eggs per gram (e.p.g.)) *versus* C-low (≤10 e.p.g.), (**b**) Canonical Correlation Analyses (CCA) of faecal microbial profiles from animals in C-high *versus* C-low, (**c)** C-high at day 0 (D0) *versus* day 2 (D2) and day 14 (D14), and (**d**) C-low at D0 *versus* D2 and D14.

Global analyses of faecal microbial composition of C-high *versus* C-low at D0 (pre-treatment), using Principal Coordinates analyses (PCoA) with Bray-Curtis distance estimates, did not show marked clustering according to FEC (Fig. 1a); however, a significant difference between these two groups was detected using supervised CCA (*P* = 0.002; F = 1.47) (Fig. 1b), whilst the effect of gender was insignificant (*P* = 0.105; F = 1.13), and on a different axis, in the same model (Supplementary Fig. S4). Additionally, the microbial profiles of  both C-high and C-low clustered separately according to sample collection time points (D0, D2 and D14) (C-high *P* = 0.001, F = 1.33; C-low *P* = 0.001, F = 1.99) (Fig. 1c,d).

### Microbial richness is reduced in animals with large parasite burdens, and increased following anthelmintic treatment

**Figure 2:**
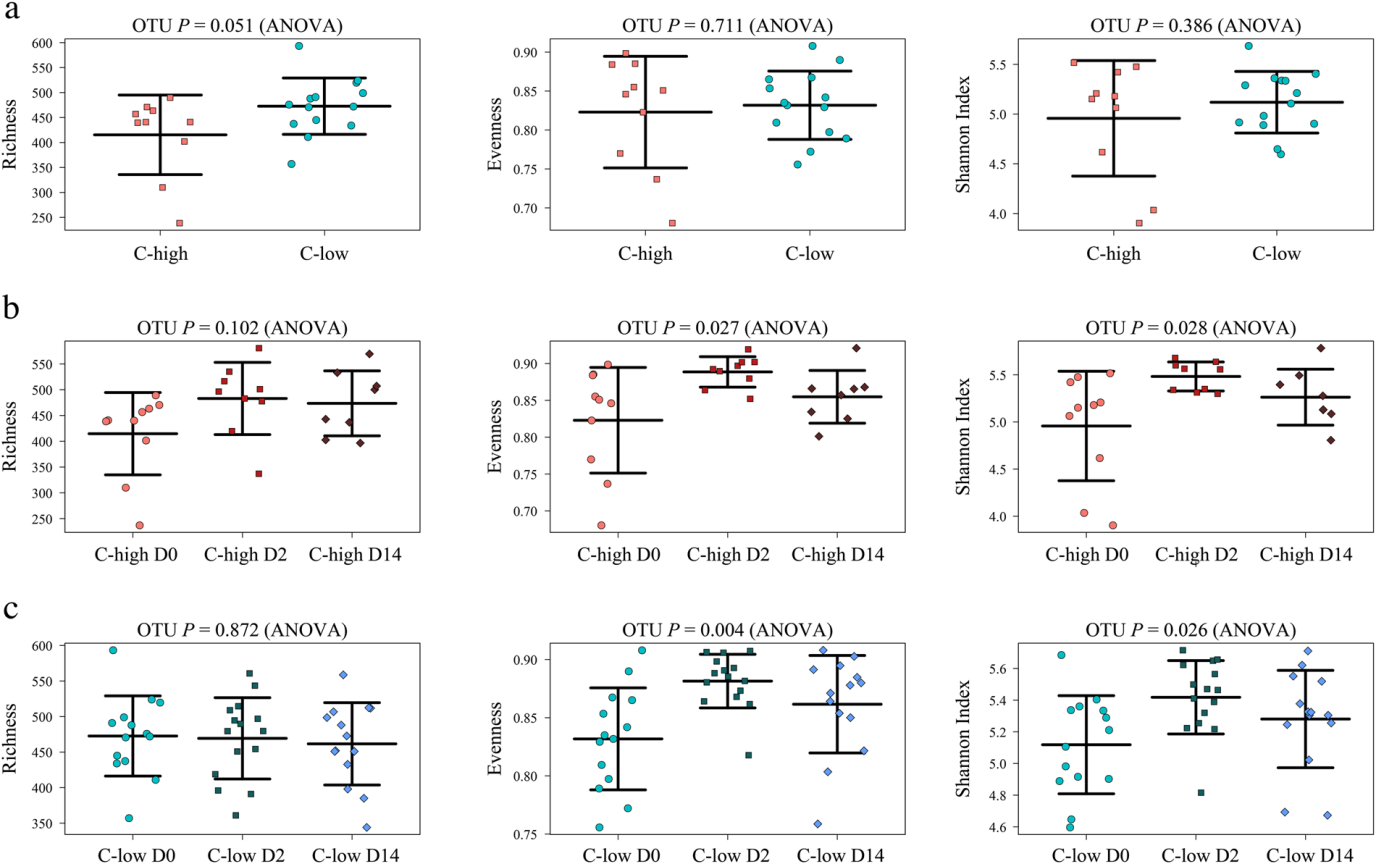
Faecal microbial richness is reduced in animals with high parasite burdens, and increased post-anthelmintic treatment. Microbial alpha diversity (based on Operational Taxonomic Unit [OTU] presence and abundance), measured by richness, evenness and Shannon index, in faecal samples from (**a**) C-high (≥100 eggs per gram (e.p.g.)) *versus* C-low (≤10 e.p.g.), (**b**) C-high at day 0 (D0) *versus* day 2 (D2) and day 14, and (**c**) C-low at D0 *versus* D2 and D14.

Microbial richness was lower in C-high at D0 (*P* = 0.05) than in C-low at the same time point (Fig. 2a). Furthermore, this parameter increased post-treatment (at D2 and D14) to levels comparable to those detected in samples from C-low at D0, although this increase was not statistically significant using ANOVA (*P* = 0.1) (Fig. 2b). No differences in faecal microbial richness were observed between samples from C-low collected at D0, D2 and D14 (*P* = 0.87) (Fig. 2c). No statistically significant differences in microbial evenness and Shannon Index were detected between C-high and C-low at D0 (Fig. 2a); however, a significant increase in microbial evenness and Shannon index was observed in both C-high and C-low at D2 and D14 when compared with samples collected at D0 (evenness C-high *P* = 0.027, C-low *P* = 0.004; Shannon Index C-high *P* = 0.028, C-low *P* = 0.026) (Fig. 2b,c). No significant differences in faecal microbial beta diversity (measured via PERMDISP) were observed between C-high and C-low, according to parasite infection burden and/or sample collection time point post-anthelmintic administration (Supplementary Fig. S5). Given the gender bias towards males in the C-high group, analysis of microbial alpha diversity was repeated for C-high and C-low at D0 with ‘gender’ as an explanatory variable; no significant difference was observed between male and female subjects (Shannon Index *P* = 0.66; evenness *P* = 0.7; richness *P* = 0.69) (Supplementary Fig. S3).

### The relationships between infection burdens, anthelmintic treatment and altered abundances of specific microbial taxa

**Figure 3:**
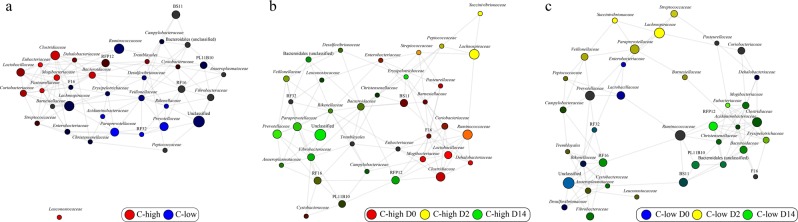
Network analyses reveal associations between faecal microbial composition, parasite infection burden, and time pre- and post-anthelmintic treatment. Pearson’s correlation network analyses showing bacterial taxa (at family level) that were positively associated to faecal samples from (**a**) C-high (≥100 eggs per gram (e.p.g.)) (in red) *versus* C-low (≤10 e.p.g.) (in blue), (**b**) C-high at Day 0 (D0) (in red), day 2 (D2) (in yellow) and day 14 (D14) (in green) post-treatment, and (**c**) C-low at D0 (in blue), D2 (in yellow) and D14 (in green). For taxa associated with multiple sample groups, the respective circle colors are mixed according to the strength of the association.

**Figure 4:**
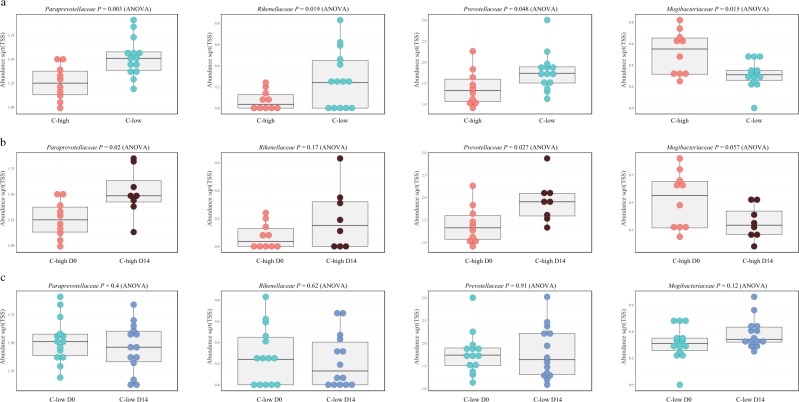
The abundances of faecal *Paraprevotellaceae*, *Rikinellaceae*, *Prevotellaceae* and *Mogibacteriaceae* prior to and following anthelmintic treatment. The relative abundances of *Paraprevotellaceae*, *Rikinellaceae*, *Prevotellaceae* and *Mogibacteriaceae* in (**a**) C-high and C-low prior to anthelmintic treatment and (**b**) C-high (≥100 eggs per gram (e.p.g.)) and (**c**) C-low (≤10 e.p.g.) prior to and following anthelmintic treatment (ANOVA).

Differences in the relative abundance of specific microbial taxa between groups were evaluated using Linear discriminant analysis Effect Size (LEfSe) and correlations between individual bacterial families, parasite infection burden and sample collection time points were represented by Pearson’s correlation network analysis (Tables 1–3; Fig. 3). At D0, Bacteroidetes were reduced in C-high compared to C-low (LDA effect size 4.66), which was largely attributable to a reduced abundance of bacteria belonging to the families *Prevotellaceae* and *Paraprevotellaceae*, and genus *Alistipes* (Family *Rikenellaceae*) (Table 1; Fig. 3a). However, in C-high, a relative expansion of bacteria belonging to the families *Mogibacteriaceae* and *Leuconostocaceae* (genus *Weissella*), and genus *Paraeggerthella* belonging to the class Clostridia (phylum Firmicutes) was observed when compared with C-low (Table 1; Fig. 3a). In the latter group, several taxa belonging to the class Clostridia, including *Anaerotruncus*, *Pseudobutyrivibrio* and Unclassified *Acidaminobacteraceae*, were increased compared with C-high (Table 1; Fig. 3a). When assessing the impact of anthelmintic treatment on the faecal microbial composition of yearlings enrolled in our study, LEfSe analysis revealed a significant decrease of bacteria belonging to the phylum Firmicutes in C-high at D14 when compared to D0, which was largely attributable to a reduction in *Mogibacteriaceae*, *Dehalobacteriaceae*, *Lactobacillaceae*, *Streptococcaceae* and *Enterococcaceae* (Table 2; Fig. 3b); in contrast, *Prevotellaceae* were increased in faecal samples collected from C-high at D14 compared with D0 (Table 2; Fig. 3b). A transient expansion of bacteria belonging to the families *Lachnospiraceae*, *Clostridiaceae* and *Succinovibrionaceae* was also observed in C-high at D2 (Table 2; Fig. 3b). In C-low, Betaproteobacteria (order) and Verrucomicrobia (phylum) were reduced and increased, respectively, at D14 when compared to D0 and D2 (Table 3; Fig. 3c). Similar to samples collected from C-high, bacteria belonging to the families *Lachnospiracaeae*, *Clostridiaceae* and *Succinivibrionaceae* were increased at D2 compared with D0 and D14, respectively (Table 3; Fig. 3c). Of the several bacterial taxa whose abundance was significantly different in samples from C-high and C-low collected pre- and post-anthelmintic treatment, four (i.e. *Mogibacteriaceae*, *Prevotellaceae*, *Paraprevotellaceae* and *Rikenellaceae*; analysed individually using ANOVA at family level) were significantly affected at D14 following the administration of ivermectin in C-high, whilst remaining unchanged in C-low at the same time point (Fig. 4).

**Table 1:** Increased abundance of *Mogibacteriaceae*, *Leuconostocaceae* and *Eubacteriaceae*, and reduced abundance of *Prevotellaceae*, *Paraprevotellaceae* and *Rikenellaceae* in faecal samples from youngstock with high parasite infection burdens.

Phylum	Class	Order	Family	Genus	Species	C-high	C-low
Bacteroidetes							
	Bacteroidia						
		Bacteroidales					
			*Prevotellaceae*				
			*Paraprevotellaceae*				
			*Rikenellaceae*	*Alistipes*			
			*Bacteroidaceae*				
				BF311			
Firmicutes	Bacilli	Lactobacillales	*Leuconostocaceae*				
				*Weissella*			
	Clostridia	Clostridiales	*Mogibacteriaceae*				
				*Mogibacterium*			
					*Mogibacterium* (unclassified)		
			*Eubacteriaceae*	*Paraeggerthella*	*Paraeggerthella hongkongensis*		
			*Lachnospiracaeae*	*Pseudobutyrivibrio*			
			*Acidaminobacteraceae*				
				Acidaminobacteraceae (unclassified)			
					Acidaminobacteraceae (unclassified)		
			*Clostridiaceae*	*Anaerotruncus*	*Anaerotruncus* unclassified		
Proteobacteria	Gammaproteobacteria	Pasteurellales					
			*Pasteurellaceae*				
	Epsilonproteobacteria	Campylobacterales	*Camplylobacteraceae*	*Campylobacter*	*Campylobacter jejuni*		
	Alphaproteobacteria	RF32					
			RF32 (unclassified)				
				RF32 (unclassified)			
	Betaproteobacteria	Burkholderiales					

Differences in relative abundance of selected microbial taxa in faecal samples from C-high (faecal egg count (FEC) > 100 eggs per gram (e.p.g.)) and C-low (FEC < 10 e.p.g.). Light grey: effect size of 3.5–4.5; dark grey: effect size >4.5.

**Table 2:** Increased abundance of Clostridiales and *Prevotellaceae*, and reduced *Lactobacillaceae* and *Mogibacteriaceae* in faecal samples from youngstock with high infection burdens following anthelmintic treatment.

Phylum	Class	Order	Family	Genus	Species	D0	D2	D14
Bacteroidetes	Bacteroidia	Bacteroidales	*Prevotellaceae*					
				*Prevotellaceae* unclassified				
					*Prevotellaceae unclassified*			
Firmicutes								
	Bacilli							
		Lactobacillales						
			*Lactobacillaceae*					
				*Lactobacillus*				
			*Enterococcaceae*	*Enterococcus*				
					*Enterococcus casseliflavus*			
			*Streptococcaceae*					
				*Streptococcus*				
		Erysipelotrichales	*Erysipelotrchaceae*	RFN20				
					RFN20 *unclassified*			
	Clostridia	Clostridiales						
			*Lachnospiraceae*					
				*Roseburia*				
					*Roseburia inulinivorans*			
				*Pseudobutyrivibrio*				
					*Pseudobutyrivibrio unclassified*			
			*Clostridiaceae*	*Clostridium*				
			*Mogibacteriaceae*					
				*Mogibacterium*				
					*Mogibacterium unclassified*			
			*Dehalobacteriaceae*	Dehalobacteriaceae unclassified				
					*Dehalobacteriaceae unclassified*			
			*Ruminococcaceae*					
				*Oscillospira*				
					*Oscillospira guillermondi*			
				*Ruminococcus*	*Ruminococcus bromi*			
				Ruminococcaceae unclassified				
Proteobacteria	Gammaproteobacteria							
		Aeromonadales						
			*Succinivibrionacae*					

Differences in relative abundance of selected microbial taxa in faecal samples from C-high (FEC > 100 egg per gram (e.p.g.)) prior to treatment (D0), and 2 (D2) and 14 (D14) days post-treatment. Light grey: effect size of 3.5–4.5; dark grey: effect size >4.5.

**Table 3:** Increased abundance of Clostridiales, Bacteroidales and Verrucomicrobia, and reduced α- and β-Proteobacteria in faecal samples from youngstock with low parasite infection burdens prior to and following anthelmintic treatment.

Phylum	Class	Order	Family	Genus	Species	D0	D2	D14
Bacteroidetes	Bacteroidia							
		Bacteroidales						
Verrucomicrobia								
	Verruco5							
		WCHB141						
			RFP12					
				RFP12 unclassified	RFP12 unclassified			
Firmicutes	Bacilli							
		Lactobacillales						
			Lactobacillaceae	*Lactobacillus*	*Lactobacillus ruminis*			
			Enterococcaceae	*Enterococcus*	*Enterococcus casseliflavus*			
				*Enterococcus*				
	Clostridia	Clostridiales	Lachnospiraceae					
				*Roseburia*				
					*Roseburia inulinivorans*			
				*Pseudobutyrivibrio*				
					*Pseudobutyrivibrio* unclassified			
			Clostridiaceae	*Clostridium*				
					Clostridium butyricum			
			Acidaminobacteraceae	*Mitsuokella*				
					*Mitsuokella multacida*			
			Eubacteriaceae	*Eubacterium*				
					*Eubacterium* unclassified			
			Eubacteriaceae					
			Ruminococcaceae					
				*Oscillospira*				
					*Oscillospira guillermondi*			
				*Ruminococcus*	*Ruminococcus bromii*			
				*Ruminococcaceae unclassified*				
				*Ruminococcus*				
					*Ruminococcus callidus*			
Proteobacteria	Gammaproteobacteria							
		Aeromonadales						
			Succinivibrionacae					
	Betaproteobacteria							
		Burkholderiales						
	Alphaproteobacteria	RF32						
			RF32 unclassified					
				RF32 unclassified				

Differences in relative abundance of selected microbial taxa in faecal samples from C-low (FEC < 10 egg per gram (e.p.g.)) prior to treatment (D0), and 2 (D2) and 14 (D14) days post-treatment. Light grey: effect size of 3.5–4.5; dark grey: effect size >4.5.

### Faecal metabolite changes associated with high- and low infection burdens and anthelmintic treatment

**Figure 5:**
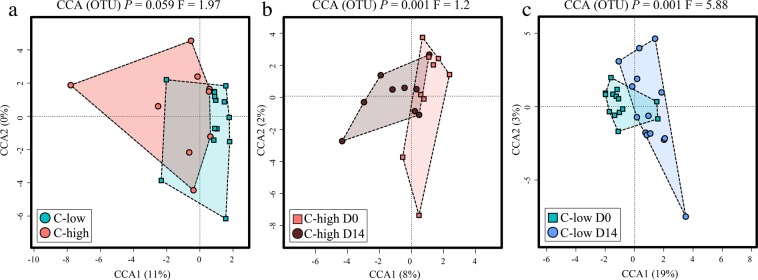
The faecal metabolic profiles of youngstock with high and low parasite infection burdens prior to and following anthelmintic treatment. Canonical Correlation Analyses (CCA) plots depicting differences between global faecal metabolic profiles of samples from (**a**) C-high (≥100 eggs per gram (e.p.g.)) (in red) *versus* C-low (≤10 e.p.g.) (in blue); (**b**) C-high at day 0 (D0) (in red) and day 14 (D14) (in brown); and (**c**) C-low at D0 (in green) and D14 (in blue).

**Figure 6:**
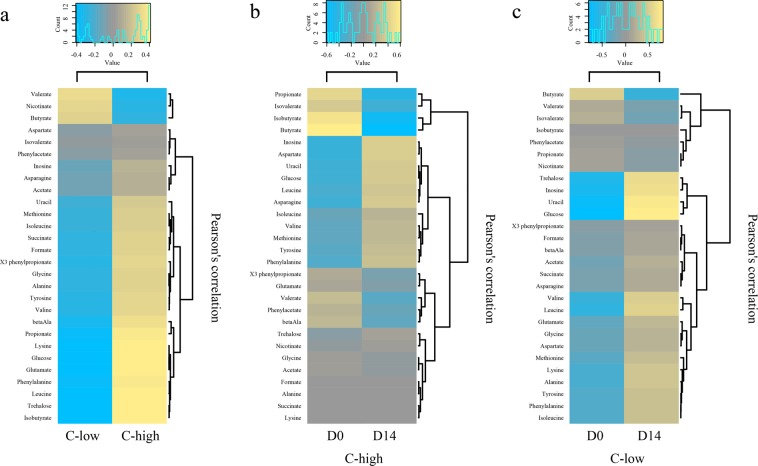
Faecal metabolic profiles of youngstock with high and low parasite infection burdens prior to and following anthelmintic treatment. Pearson’s correlation heatmaps depicting differences in the relative abundances of faecal metabolites between (**a**) C-high (≥100 eggs per gram (e.p.g.)) and C-low (≤10 e.p.g.) at day 0 (D0); (**b**) C-high at D0 and day 14 (D14); and (**c**): C-low at D0 and D14.

A total of 28 faecal metabolites in all samples from C-high and C-low collected at D0 and D14 were identified and quantified by Proton Nuclear Magnetic Resonance (^1^H-NMR). These were subjected to PCoA (Supplementary Fig. S6) and CCA; the latter allowed us to identify differences between the faecal metabolomes of C-high and C-low at D0 (*P* = 0.059; F = 1.97) (Fig. 5a). Pearson’s correlation heatmap analyses demonstrated increased abundances of isobutyrate, trehalose, leucine, phenylalanine, glutamate, glucose, lysine and propionate, and decreased nicotinate, valerate and butyrate, in C-high *versus* C-low (Fig. 6a); however, these differences were not statistically significant according to ANOVA following False Discovery Rate (FDR) correction for multiple testing (*P* > 0.05 for all metabolites) (Supplementary Table S2). CCA also detected a difference between the faecal metabolomes of C-high at D0 and D14 (*P* = 0.001; F = 1.2), that however, for individual metabolites, was not statistically significant using ANOVA following FDR correction (*P* > 0.05 for all metabolites) (Fig. 5b; Supplementary Table S3); nevertheless, a relative reduction in the short chain fatty acids (SCFAs) butyrate, isobutyrate and propionate was observed in samples from this group at D14 *versus* D0 *via* Pearson’s correlation heatmaps (Fig. 6b). Conversely, in addition to a greater effect size according to CCA (*P* = 0.001; F = 5.88), clear differences were detected between the faecal metabolites identified in samples from C-low at D0 and D14 (Fig. 5C; Supplementary Table S4). In particular, glucose, uracil, inosine, trehalose, leucine, butyrate and valine were more abundant in samples collected at D14 when compared to D0 (ANOVA; *P* < 0.001, *P* < 0.001, *P* = 0.001, *P* = 0.003, *P* = 0.022, *P* = 0.041 and *P* = 0.044, respectively) (Fig. 6c; Supplementary Table S4). Partial Least Squares (PLS) analysis of metabolite and bacterial OTU data obtained from faecal samples at D0, with ‘metabolites’ as the dependent matrix and ‘bacterial OTUs’ as the independent matrix, did not identify any significant association between these two datasets (*P* = 0.96)

## Discussion

In this study, acute helminth infections in a cohort of equine youngstock were associated with significant alterations in gut microbial profiles and diversity, which were reversed following parasiticide treatment. The faecal microbial profiles of the yearlings in our study were in line with those described previously for managed equines^54,67,69,72,73^, with the predominance of OTUs assigned to Bacteroidetes (42.3%) and Firmicutes (42.1%); nevertheless, a greater interindividual variation in taxonomic composition was observed in this group of animals when compared to a cohort of adult broodmares hosted on the same farm^40^. This discrepancy may be suggestive of a ‘developing microbiome’ in these young animals. Indeed, a recent study in human infants demonstrated the occurrence of three stages of microbial development in the GI tract during early life, which include an early phase characterised by rapid changes in the core GI microbiota over time, an intermediate transitional phase characterised by fewer changes over time, and a final and stable ‘adult’ microbial profile^4^. In support of our findings, a previous study conducted in equine youngstock demonstrated that, although gut microbiome re-modelling occurs mostly during the first 60 days of life, the gut microbial profiles of horses of 9 months of age is still significantly different from those of adult animals^7^. Of note, environmental and pathogenic stimuli which impact on the order of colonisation of the GI tract during the developmental phase may have significant repercussions on adult microbiome composition and homeostasis^1,28,74^. Hence, given the known roles of the gut microbiome in immune regulation and metabolism, it is likely that the alterations in faecal microbiome composition of equine youngstock observed in association with GI helminth infections may result in long-term implications for animal health and wellbeing.

Indeed, the abundance of several bacterial taxa with key roles in metabolism and immune-regulation differed significantly between C-high and C-low. For instance, bacterial families belonging to the classes Clostridia and Bacteroidetes, were increased and decreased, respectively, in C-high when compared to C-low prior to anthelmintic treatment. Of note, several differences in faecal microbial profiles observed between groups before treatment were reversed following anthelmintic administration; for instance, bacteria belonging to the families *Mogibacteriaceae* (phylum Firmicutes; class Clostridia), *Prevotellaceae* (phylum Bacteroidetes; class Bacteroidetes) were similar in abundance in faecal samples from C-low and C-high by D14. A reduction in Bacteroidetes (genera *Prevotella* and *Parabacteroides*) was also observed in mice experimentally infected with the murine whipworm, *T*. *muris*^36^. Members of the family *Prevotellaceae* are commensals of mammalian mucosal surfaces, and are known potential pathobionts, particularly associated with oral infections^75^. *Prevotella* spp. have been demonstrated to promote pro-inflammatory Th17-mediated immune responses^75,76^; thus, a decreased abundance of this bacterial family in the presence of GI helminth infections could be linked to the known immune-regulatory properties of these parasites^77^. Indeed, a reduction in Th17-inducing segmented filamentous bacteria has been previously reported in association with *Nippostrongylus brasiliensis* infection in a rodent model^34^, indicating that this may be a common immune-modulatory mechanism in GI helminth infections. Nevertheless, in herbivores, *Prevotellaceae* also play a key role in the break-down of indigestible fibres^78,79^; moreover, in a study by Houlden *et al*.^36^, an observed increase in long chain fatty acids in the faeces of *T*. *muris* infected mice was attributed to inefficient breakdown of plant fibres as a result of reduced abundance of *Prevotella* and *Parabacteroides*. This dichotomy of function in *Prevotella* spp. has been well described^80^, and demonstrates that, whilst *Prevotella* are commensal bacteria with an important metabolic role, they can be pro-inflammatory in association with mucosal pathology. It is also worth reporting that, in contrast to our findings, a positive association between helminth infections and *Prevotella* spp. abundance was observed in the abomasum of 3 mo small ruminants colonised by the GI helminth *H*. *contortus*^46^. Whilst this apparent discrepancy may be linked to fundamental differences between the host:parasite systems under investigation and infection sites (stomach *versus* large intestine), this data calls for further investigations of the mechanisms governing the interactions between GI helminths and *Prevotella* spp. and their implications for the developing microbiome of young vertebrates.

Within the phylum Firmicutes, a number of families belonging to class Clostridia were significantly more abundant in the faecal microbiota of C-high when compared to C-low at D0, according to LEfSe (*Mogibacteriaceae* and *Eubacteriaceae*) and network (*Dehalobacteriaceae* and *Clostridiacae*) analyses. Clostridia have been frequently implicated in studies of helminth-microbiome interactions, with increased abundances being reported in the gut of mice experimentally infected with *T*. *muris*^36,81,82^, and in association with mixed GI helminth infections in humans^82^. Clostridia are known to exert immune-regulatory functions, since they produce SCFAs with anti-inflammatory properties, including butyrate^83,84^. Hence, the increased abundances of *Mogibacteriaceae* and other *Clostridiaceae* observed in the faecal microbiota of youngstock with heavy parasite burdens could represent a mechanism by which helminths suppress host immune responses, thus reducing pathology and facilitating the establishment of chronic infections^83^. However, *Mogibacteriaceae* are not butyrate producers^85^; in addition, metabolomic analysis of faecal samples collected in this study revealed lower levels of butyrate in faeces from C-high when compared to C-low prior to anthelmintic treatment. In accordance with this observation, a recent study investigating the faecal metabolome of human volunteers with chronic infections by *Strongyloides stercoralis* also detected lower levels of butyrate in samples from parasite-colonised individuals^42^. These data indicate that, whilst increases in SFCAs have been described previously in association with GI helminth infection^83^, this link may not be applicable to all host-parasite systems. Of note, Clostridia belonging to the family *Mogibacteriaceae* have also been associated with GI inflammation in periodontal disease^86^, and hence their increased abundance in animals with higher infection levels in this study may suggest a role for these bacteria in the onset of mucosal pathology. On the other hand, increased levels of *Mogibacteriaceae* have also been identified as a biomarker of health in studies comparing subjects with inflammatory bowel disease and irritable bowel disease, to healthy controls^87–89^. These apparent contradictions are analogous to those described above for *Prevotella* spp., and highlight the need for mechanistic studies to unravel the complex function of microbial species within the gut flora colonising different hosts, and in various disease states.

Amongst other bacterial taxa with well-known immune-modulatory functions, those belonging to the order *Lactobacillales* (phylum Firmicutes, class Bacilli), were also expanded in the faecal microbiota of C-high *versus* C-low at D0, and bacteria of the family *Lactobacillaceae* were significantly reduced following anthelmintic treatment in the former group. The existence of a mutualistic association between *Lactobacillaceae* and parasitic helminths has been hypothesised based on experimental evidence obtained from murine models of infection with the intestinal nematode *Heligmosomoides polygyrus*^38,90^. Indeed, in a key study by Reynolds *et al*.^90^, *H*. *polygyrus* infections were associated with significantly expanded populations of *Lactobacillaceae* in the gut of colonised mice; in addition, oral administration of *L*. *taiwanensis* prior to helminth exposure was followed by the onset of T regulatory cell mediated immune responses and significantly increased worm burdens^91^. Based on this knowledge, it is tempting to speculate that similar relationships may exist between cyathostomin parasites and equine hosts. Moreover, the increased abundance of bacterial taxa with immune-modulatory roles in animals with heavy burdens of helminth infections may alter the susceptibility of the equine hosts to colonisation by other, ‘opportunistic’ pathogens. Indeed, potential pathobionts such as *Campylobacter jejuni* and *Pasteurellaceae* were expanded in animals with high FEC, and reflected data collected in previous investigations of helminth-infected horses and pigs^92,93^. In humans and rodents, *Lactobacillaceae* are also generally considered to have positive health benefits for the host^94^; however, the beneficial effects of *Lactobacillaceae* to the health and homeostasis of the equine GI microbiota is yet to be demonstrated^54,95^. A recent review of data collected from equine gut microbiome studies has highlighted the roles of other bacterial taxa, such as *Lachnospiraceae* (phylum Firmicutes, class Clostridia), in supporting intestinal health^54^. In particular, increased abundances of OTUs assigned to the family *Lachnospiraceae* have been reported in the faecal microbiome of healthy horses when compared with animals with colitis^51,68^; furthermore, ponies with innate resistance to helminth infection, and consequently lower parasite burdens, have been shown to harbour larger populations of this bacterial family^93^. Accordingly, in our study, the genus *Pseudobutyrivibrio* (family *Lachnospiraceae*) was significantly more abundant in the faecal microbiome of C-low *versus* C-high prior to treatment. Together, this data points towards a detrimental effect of acute cyathostomin infections on the development and maturation of the equine gut microbiome, an effect which is highly likely to have repercussions on susceptibility to a range of infectious and non-infectious diseases^1,27,93,96^. In support of this hypothesis, faecal microbial richness (a measure of microbial alpha diversity and a proxy of gut ‘health’^29,66,97–99^) was significantly lower in samples from C-high when compared to C-low prior to treatment. Acute helminth infections have been frequently associated with decreased alpha diversity of microbial gut populations^36,42,43^ and attributed primarily to alterations of the GI microenvironment in response to mounting immune reactions against the invading parasites. Nevertheless, these data contrast with our previously published work in which we reported a trend towards increased alpha diversity in the gut microbiome of chronically helminth-infected adult horses^40^. Differences between the systems investigated (i.e. acutely *versus* chronically infected horses) may provide a possible explanation, whereby infections of immunologically naïve animals and subsequent pro-inflammatory responses are accompanied by a significant decrease in gut microbial diversity; the latter may be restored (or increased; cf.^39,42,100^) during chronic helminth infections, due to a synergic effect of host adaptive immune responses and immune-suppressive properties of helminth parasites^101^ that contributes to the dampening of local inflammation^102^.

Given the impact of helminth infections on the composition and diversity of the youngstock gut microbiome, we sought to investigate the associations between such changes and the composition of the faecal metabolome. A multivariate analyses of faecal metabolite levels in samples from C-high and C-low prior to and following ivermectin administration revealed only moderate differences between the faecal metabolic profiles of these two groups. A greater abundance of the SCFA isobutyrate, the amino acids leucine, lysine and phenylananine, and the products of carbohydrate breakdown glucose and trehalose were observed in samples from C-high *versus* C-low prior to ivermectin administration. Of note, increases in selected amino acid abundance have also been described in the faecal metabolome of both mice^36^ and humans infected by GI helminths^42^. This consistent observation may indicate a reduction in the absorption of the products of microbial metabolism, for example, as a consequence of ongoing intestinal inflammation caused by helminth colonisation. This hypothesis is supported by the lack of significant correlations between the abundance of specific gut bacteria and metabolites detected by PLS analysis, which might indicate that host-related factors, such as malabsorption, might have been responsible for the observed changes. In the context of livestock management, a reduction in amino acid absorption is likely to have considerable implications for animal performance and production. Thus, the relationships between metabolite production and absorption should be further investigated via metabolomics analyses of faeces and other biofluids (e.g. urine and blood) of equines and other livestock species infected by parasitic helminths^103^. Administration of anthelmintics to C-high did not result in significant alterations of concentrations of faecal metabolites detected before treatment, suggesting that any malabsorptive component of the observed increase in amino acids and glucose, did not resolve over the time-scale of the study. In contrast, concentrations of glucose, uracil, inosine and trehalose in faecal samples from C-low were significantly increased post-treatment. As these animals did not harbour significant parasite burdens prior to anthelmintic treatment, this finding supports a possible role of other environmental factors, such as grass quality and/or anthelmintic administration, on faecal metabolite and gut bacteria abundance over time.

### Concluding remarks

Data from both bacterial 16S rRNA sequences and ^1^H-NMR analyses of faecal samples from equine youngstock with high *versus* low parasite burdens revealed a number of helminth-associated perturbations to GI microbial composition and metabolism, with likely repercussions on medium- to long-term host susceptibility to a range of infections and diseases which should be further investigated. In particular, we advocate for in-depth studies of the impact that early colonisation of young production animals by parasites (e.g. calves, lambs and piglets) exerts on host gut microbial composition and metabolism, given the likely severe economic impact of alterations of the gut homeostasis of these species. In addition, data from our study calls for further explorations of the role/s that parasite-associated changes in gut microbiota may play in the immunopathology of helminth infections in children from endemic areas, and their susceptibility to other parasitic, bacterial and viral infectious agents in areas of poor sanitation.

## Methods

### Ethics statement

This study was approved and carried out in strict accordance and compliance with the guidelines of the Institutional Ethical Review Committee, Department of Veterinary Medicine, University of Cambridge, UK (Ref. No. CR190). Written informed consent was obtained from the stud farm from which study samples were collected.

### Study population and diagnostic procedures

A cohort of TB yearlings was recruited from a stud farm in eastern England, UK. The stud hosts ~50 yearlings each year, which are kept at pasture in groups of 2–8 across 486 hectares. Routine control of parasite infections in the stud relies on administration of targeted anthelmintic treatments (with ivermectin and fenbendazole), based on evaluation of individual parasite burdens (inferred by FEC, that consist in counting the number of parasite e.p.g. of faeces) at 2 monthly intervals. In addition, praziquantel is administered to each yearling three times a year for tapeworm control, whilst a single moxidectin treatment is administered in late November for control of cyathostomins. Samples used in this study were collected in April 2017; all yearlings had received ivermectin (0.2 mg/kg) and fenbendazole (10 mg/kg) in February 2017, and praziquantel (1.5 mg/kg) in March 2017. A total of 53 TB yearlings, between 12–16 months of age at the time of sampling, were screened for infection by cyathostomins. Briefly, duplicate faecal samples were collected from all horses on D0; aliquots of each sample were subjected to (i) FEC analysis using a centrifugal floatation technique sensitive to one e.p.g.^104^, and (ii) screening for infections with the common equine cestode *Anoplocephala perfoliata* using a double sugar flotation technique^105^, and (iii) larval culture and microscopic examination to screen for infections with large strongyle nematodes, e.g. *Strongylus vulgaris*. Horses were recruited in the study if they satisfied the following criteria: (i) FEC of ≥100 e.p.g. (=C-high) or ≤10 e.p.g. (C-low) in duplicate faecal samples at D0; (ii) negative for co-infections with other GI helminths (i.e. *Strongylus* spp., *Parascaris equorum*, *Strongyloides westeri*, *A*. *perfoliata*) and no history of or clinical signs of infection by *Oxyuris equi*; (iii) no antibiotic treatment for at least 2 months prior to sampling. Of the 53 horses screened, 23 matched these criteria, of which 9 were enrolled into the C-high group, and 14 into C-low (Supplementary Table S1). A power calculation was performed based on a bacterial 16S rRNA amplicon sequence dataset from a previous study conducted using samples from equines on the same farm^40^. A Wilcoxon-Mann-Whitney test for comparing OTU-specific abundances between two samples, as described by Mattiello *et al*.^106^ (https://fedematt.shinyapps.io/shinyMB), demonstrated that the current study with 23 samples had sufficient power (0.81) to detect an effect size of 2, in at least 20 OTUs between C-high and C-low.

### Anthelmintic treatment and sampling

Individual, freshly voided, faecal samples were collected from the centre of the faecal mass from all C*-*high and C-low animals at D0. Immediately following sample collection, an anthelmintic treatment (Eqvalan: ivermectin 0.2 mg/kg) was administered to each animal. Sampling was repeated as above at D2 and D14. A 100 g aliquot of each faecal sample was snap frozen, transported to the laboratory and stored at −80 °C within 2 h of collection, prior to genomic DNA extraction, high-throughput sequencing of the bacterial 16S rRNA gene and metabolite extraction; the remainder was kept fresh and subjected to FEC analysis as described above.

### DNA extractions and bacterial 16S rRNA gene Illumina sequencing

Previously published^40,42^ bacterial 16S rRNA high-throughput sequencing protocols and bioinformatics analyses of sequence data were adapted for this study. Briefly, genomic DNA was extracted from each faecal sample, as well as from five negative ‘blank’ (=no DNA) controls, using the PowerSoil® DNA Isolation Kit (Qiagen, Carlsbad, CA, USA), according to the manufacturers’ instructions. High-throughput sequencing of the V3-V4 hypervariable region of the bacterial 16S rRNA gene was performed by Eurofins Genomics on an Illumina MiSeq platform according to the standard protocols with minor adjustments. Briefly, the V3-V4 region was PCR-amplified using universal primers, that contained the adapter overhang nucleotide sequences for forward (TACGGGAGGCAGCAG) and reverse primers (CCAGGGTATCTAATCC). Amplicons were purified using AMPure XP beads (Beckman Coulter) and set up for the index PCR with Nextera XT index primers (Illumina). The indexed samples were purified using AMPure XP beads (Beckman Coulter) and quantified using the Fragment Analyzer Standard Sensitivity NGS Fragment Analysis Kit (Advanced Analytical) and equal quantities from each sample were pooled. The resulting pooled library was quantified using the Agilent DNA 7500 Kit (Agilent), and sequenced using the v3 chemistry (2 × 300 bp paired-end reads, Illumina).

### Bioinformatics and statistical analyses of 16S rRNA sequencing data

Raw paired-end Illumina reads were trimmed for 16S rRNA gene primer sequences using Cutadapt (https://cutadapt.readthedocs.org/en/stable/) and sequence data were processed using the Quantitative Insights Into Microbial Ecology 2 (QIIME2-2018.4; https://qiime2.org) software suite^107^. Successfully joined sequences were quality filtered (Read cut-off: 17; 286 and 17; 255 for forward and reverse, respectively), dereplicated, chimeras identified, and paired-end reads merged in QIIME2 using DADA2^108^. A phylogenetic tree was generated for diversity analysis, followed by calculation of alpha and beta diversity metrics using the ‘core-metrics-phylogenetic command’ in QIIME2. Sequences were assigned to taxonomy using the feature classifier: Greengenes 13_8 99% OTUs full-length sequences. A feature table with the assigned taxonomy was exported from QIIME2 alongside a weighted UniFrac distance matrix for downstream biostatistical analysis. Statistical analyses were executed using the Calypso software^109^ (cgenome.net/calypso/); total sum scaling (TSS) normalisation was applied, followed by square route transformation to account for the non-normal distribution of taxonomic counts data. Microbial community profiles were ordinated using PCoA (Bray-Curtis distances) and supervised CCA including infection status and sample collection time point as explanatory variables. Differences in bacterial alpha diversity (richness, evenness and Shannon Index) between study groups (C-high and C-low at D0; as well as D0, D2 and D14 for each C-high and C-low) were evaluated based on rarefied data (read depth of 25,868) using ANOVA. Differences in beta diversity (weighted UniFrac distances) were measured using PERMDISP^110^. Differences in the abundances of individual microbial taxa between groups were assessed using the LEfSe workflow^111^, accounting for the paired nature of samples pre- and post-anthelmintic treatment. Furthermore, networks of correlation were constructed using the Calypso software^109^ to identify clusters of co-occurring bacteria based on their association with helminth infection status. Taxa and explanatory variables were represented as nodes, taxa abundance as node size, and edges represented positive associations, while nodes were coloured according to infection status. Taxa abundances were associated with infection status using Pearson’s correlation and nodes were then coloured based on the strength of this association. Networks were generated by first computing associations between taxa using Pearsons’s rho and the resulting pairwise correlations were converted into dissimilarities and then used to ordinate nodes in a two-dimensional plot by PCoA. Therefore, correlating nodes were located in close proximity and anti-correlating nodes were placed at distant locations in the network.

### Metabolite extraction

Metabolites were extracted from 200 mg aliquots of each faecal sample using a methanol–chloroform–water (2:2:1) procedure, as described previously^42^. In particular, 600 μl of methanol–chloroform mix (2:1 v:v) were added, samples were homogenised using stainless steel beads and sonicated for 15 min at room temperature. 200 μl each of chloroform and water were added, samples were centrifuged and the separated aqueous and lipid phases were collected. The procedure was repeated twice, and the aqueous fraction from each extraction were pooled. The aqueous fraction was dried in a vacuum concentrator (Concentrator Plus, Eppendorf).

### ^1^H-NMR analysis of aqueous extracts

Protocols of ^1^H-NMR metabolite analysis have been described previously^42^. Briefly, the dried aqueous fractions were re-dissolved in 600 μl D_2_O, containing 0.2 mM sodium-3-(tri-methylsilyl)-2,2,3,3-tetradeuteriopropionate (TSP) (Cambridge Isotope Laboratories, MA, USA) as an internal standard and phosphate buffer (40 mM NaH_2_PO_4_/160 mM Na_2_HPO_4_). The samples were analysed using an AVANCE II + NMR spectrometer operating at 500.13 MHz for the ^1^H frequency and 125.721 MHz for the ^13^C frequency (Bruker, Germany) using a 5 mm TXI probe. The instrument is equipped with TopSpin 3.2. Spectra were collected using a solvent suppression pulse sequence based on a one-dimensional nuclear Overhauser effect spectroscopy (NOESY) pulse sequence to saturate the residual 1 H water signal (relaxation delay = 2 s, t1 increment = 3 us, mixing time = 150 ms, solvent pre-saturation applied during the relaxation time and the mixing time). One hundred and twenty-eight transients were collected into 16 K data points over a spectral width of 12 ppm at 27 °C. In addition, representative samples of each data set were also examined by two-dimensional Correlation Spectroscopy (COSY), using a standard pulse sequence (cosygpprqf) and 0.5 s water presaturation during relaxation delay, 8 kHz spectral width, 2048 data points, 32 scans per increment, 512 increments. Peaks were assigned using the COSY spectra in conjunction with reference to previous literature and databases and the Chenomx spectral database contained in Chenomx NMR Suite 7.7 (Chenomx, Alberta, Canada). 1D-NMR spectra were processed using TopSpin. Free induction decays were Fourier transformed following multiplication by a line broadening of 1 Hz, and referenced to TSP at 0.0 ppm. Spectra were phased and baseline corrected manually. The integrals of the different metabolites were obtained using Chenomx. Metabolites were normalised to faecal dry matter, total area and differential abundance of metabolites between samples from C-high and C-low, at D0 and D14. Faecal metabolite abundances from each sample were ordinated by PCoA according to infection status (C-high and C-low) and sample collection time point (D0 and D14). Predicted associations among metabolites identified in the faecal metabolome of each sample group were also identified by Pearson’s correlation heatmaps in Calypso^109^ (cgenome.net/calypso/). In particular, heatmaps were constructed to identify associations between metabolite abundance and infection status (i.e. C-high and C-low) and time point pre- and post-treatment (i.e. D0 and D14). Differences in individual metabolite abundance between groups were evaluated for statistical significance using ANOVA with FDR correction for multiple comparisons. In order to identify linear correlations between metabolites and bacterial OTUs identified in faecal samples, PLS analysis was performed on data from all samples at D0 (pre-treatment) in Simca-P v15, with metabolites as the dependent and OTU as the independent matrix, respectively.

## Supplementary information

Supplementary Information

## Data Availability

The raw 16s rRNA and ^H^NMR data, metadata and QIIME2 feature table are available at Mendeley Data (10.17632/95m8sfd3kt.1).
