# Supplementary material for: Dysbiosis associated with acute helminth infections in herbivorous youngstock – observations and implications
Source: Sci Rep. 2019 Jul 31;9:11121. doi: 10.1038/s41598-019-47204-6 (PMC6668452; doi:10.1038/s41598-019-47204-6)
Supplement: Supplementary file 1 — Supplementary Information [file 41598_2019_47204_MOESM1_ESM.pdf]

Dysbiosis associated with acute helminth infections in herbivorous youngstock – observations and implications

Supplementary Information

Laura E. Peachey[1,2], Cecilia Castro[3], Rebecca A. Molena[1], Timothy P. Jenkins[1], Julian L. Griffin[3], Cinzia Cantacessi[1]

[1] Department of Veterinary Medicine, University of Cambridge, Cambridge, United Kingdom.

[2] Bristol Veterinary School, University of Bristol, Langford, United Kingdom.

[3] Department of Biochemistry, University of Cambridge, Cambridge, United Kingdom.

Correspondence and requests for materials should be addressed to L.E.P. (lep41@cam.ac.uk or laura.peachey@bristol.ac.uk) or C.C. (cc779@cam.ac.uk)

Supplementary Fig. S1: | Rarefaction analyses indicated sufficient sampling depth. Rarefaction curves demonstrating the relationship between sampling depth and microbial richness in each faecal sample from C-high (≥ 100 eggs per gram (e.p.g.)) and C-low (≤10 e.p.g.).

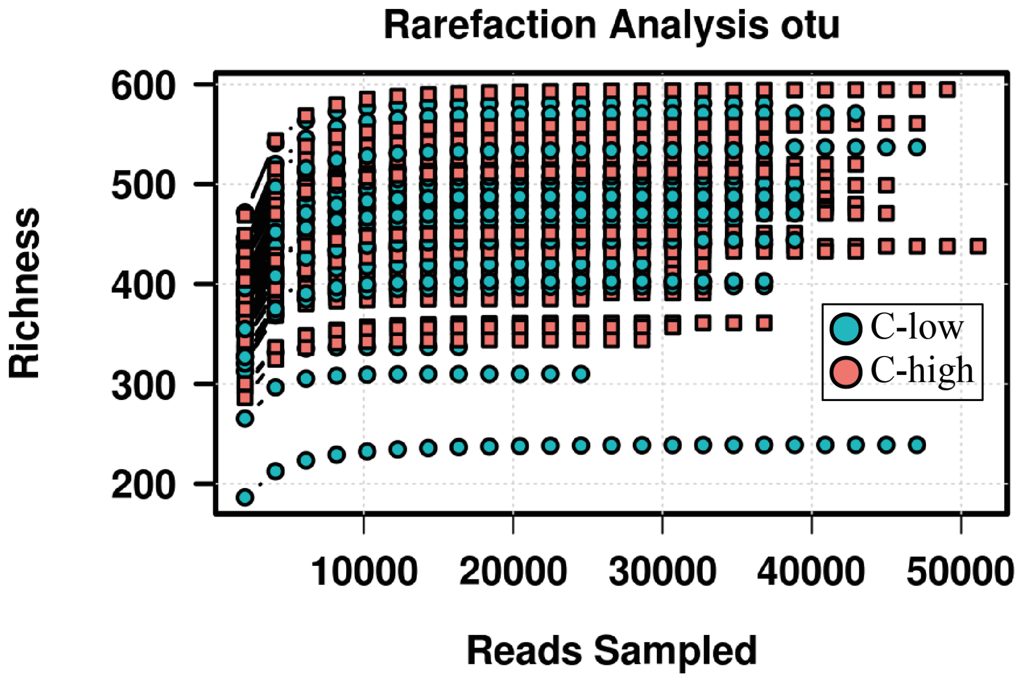

Supplementary Fig. S2: | Taxonomic visualisation demonstrates a predominance of the phyla Firmicutes and Bacteroidetes. The relative proportions of individual bacterial phyla in each faecal sample from animals in C-high (≥100 eggs per gram (e.p.g.)) and C-low (≤10 e.p.g.) at day 0 (D0); C-high at D0, day 2 (D2) and day 14 (D14) post-anthelmintic treatment; and C-low at D0, D2 and D14.

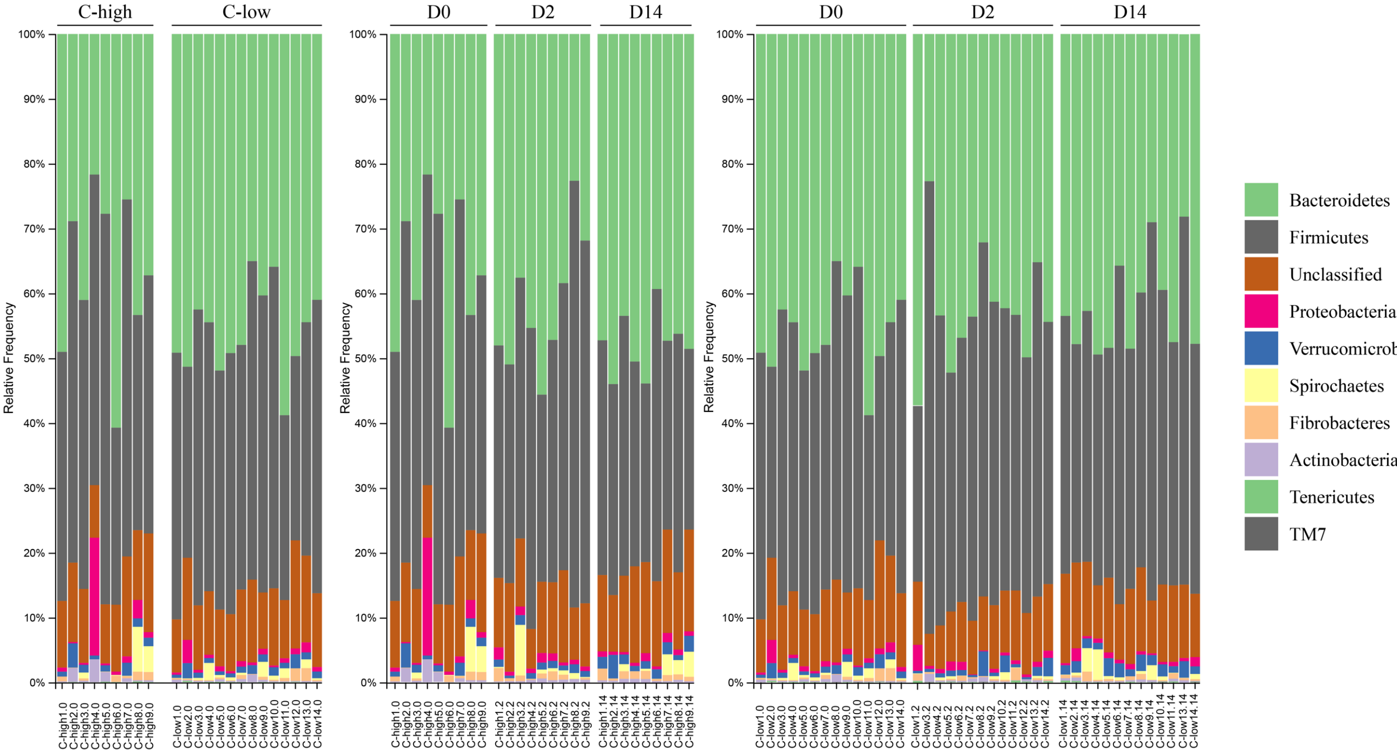

Supplementary Fig. S3: | Gender does not impact on global faecal microbial or metabolite composition or diversity. Comparison of faecal microbial and metabolite composition and diversity according to gender of equines included in this study by **a**: comparison of faecal microbial composition between female (red) and males (blue) horses at Day 0 (D0) by Canonical Correspondence Analyses (CCA); **b**: comparison of faecal microbial alpha diversity (measured by Shannon Index, evenness and richness) between female and male horses at D0; **c**: Pearson's Correlation Network analyses depicting bacterial taxa (at Family level) that correlate with faecal samples from either female (red) or male (blue) horses at D0; **d**: comparison of global faecal metabolite levels between female (red) or male (blue) horses at D0.

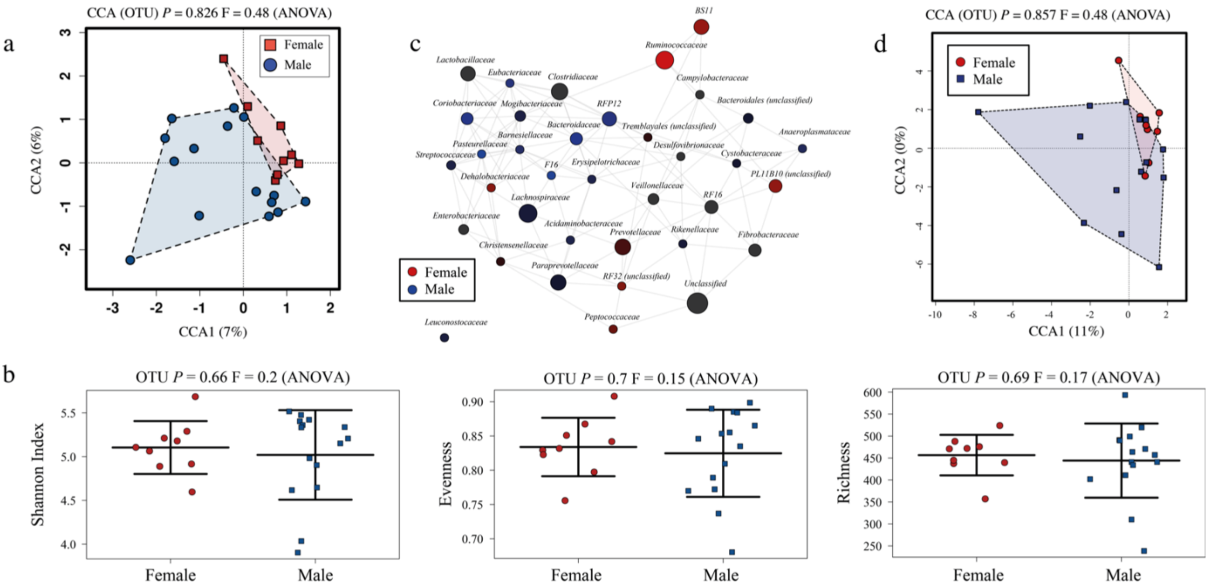

Supplementary Fig. S4: | Gender does not impact significantly on faecal microbial composition pre-treatment. Biplot of Canonical Correspondence Analysis (CCA) showing the relative effects of gender (*P*=0.105) and infection (i.e. between C-high and C-low) (*P*=0.002) on global microbial composition (measured by Bray Curtis distance estimates).

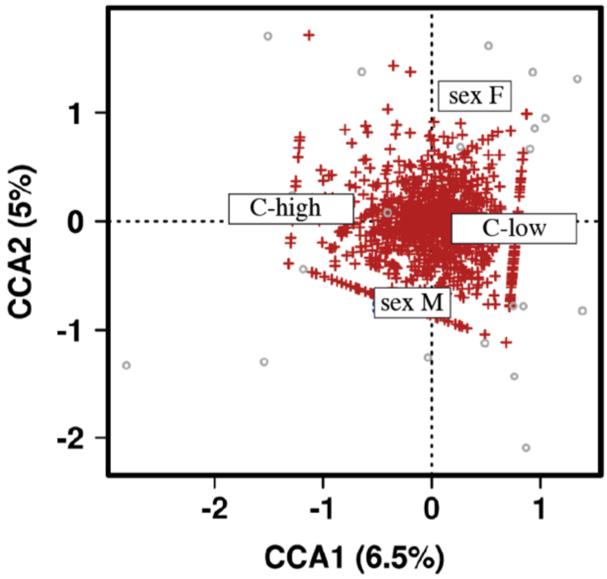

Supplementary Fig. S5: | Analysis of beta diversity showed no significant differences between groups. Comparison of microbial beta diversity (PERMDISP2) between **a**: C-high (≥100 eggs per gram (e.p.g.)) *versus* C-low (≤10 e.p.g.) at day 0 (D0); **b**: C- high at D0, day 2 (D2) and day 14 (D14), and **c**: C-low at D0, D2 and D14.

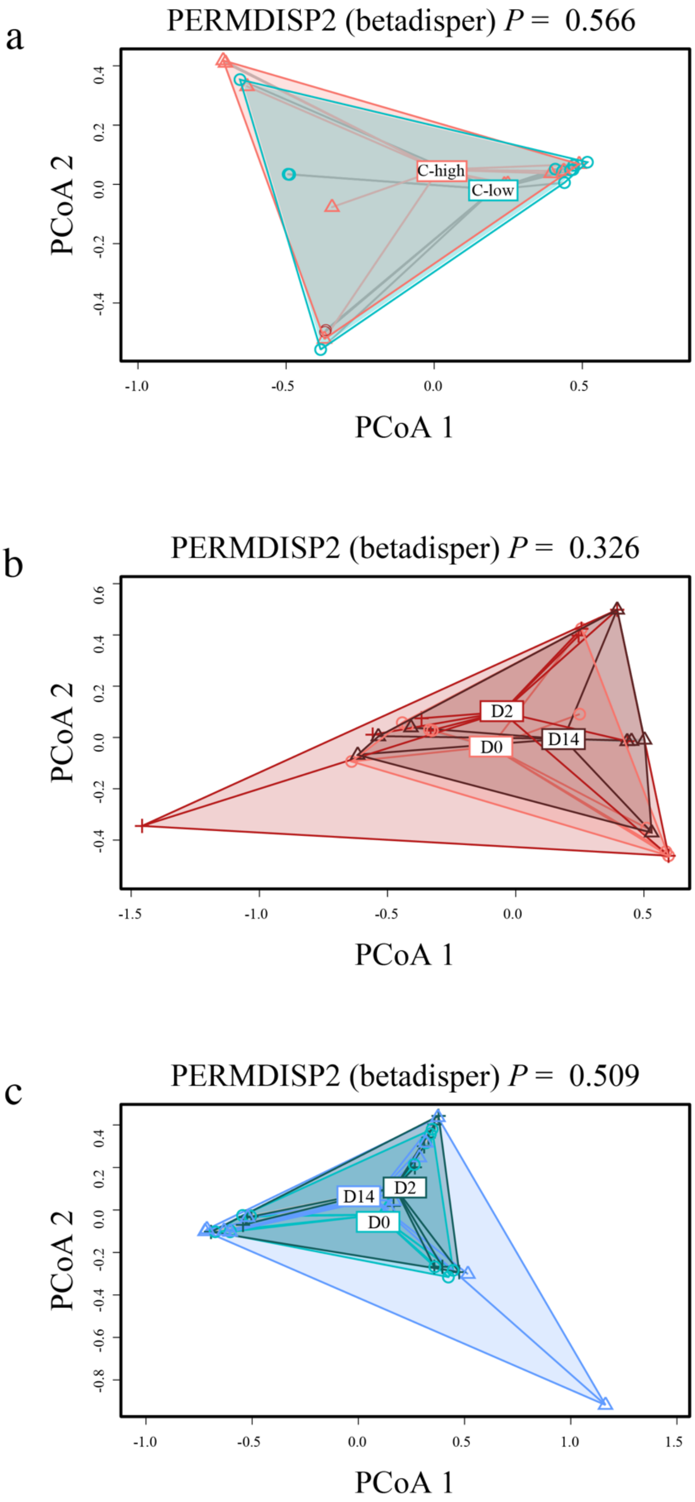

Supplementary Fig. S6: | **Principal Coordinates Analyses does not show marked differences in faecal metabolite profiles between groups.** Principal Coordinates Analyses (PCoA) plots displaying differences between the global faecal metabolic profiles of **a**: C-high (≥100 eggs per gram (e.p.g.)) (in red) *versus* C-low (≤10 e.p.g.) (in blue); **b**: C-high at Day 0 (D0) (in red) and day 14 (D14) (in brown) post-anthelmintic treatment, and **c**: C-low at D0 (in green) and D14 (in blue).

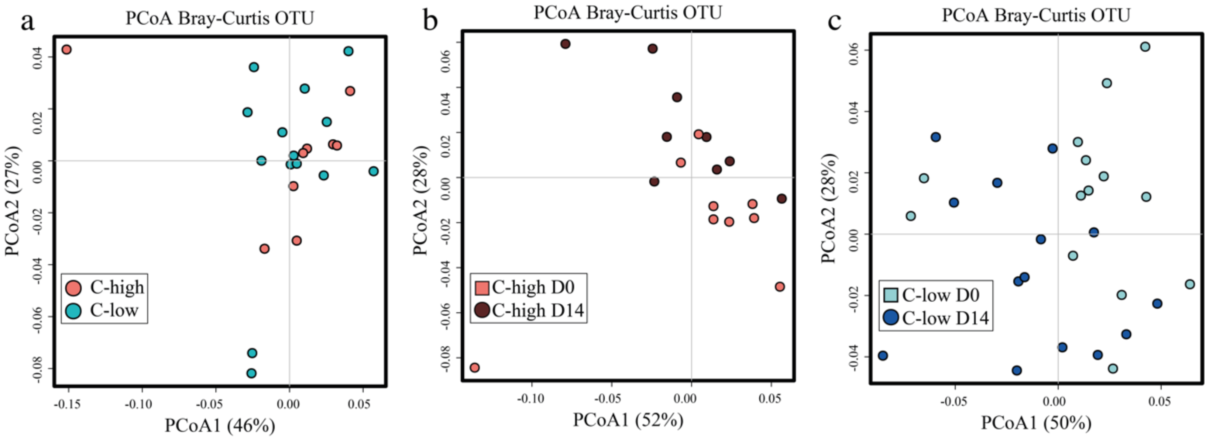

Supplementary Table S1: | Animals were assigned to groups according to their parasite infection burdens, and parasites were eliminated following anthelmintic treatment. Faecal egg counts (FEC) recorded from C-high (FEC ≥100 eggs per gram, e.p.g.) and C-low (FEC ≤ 10 e.p.g.) youngstock, over two consecutive samplings performed pre-anthelmintic treatment on Day 0 (D0), as well as at 2 and 14 days post-treatment (D2 and D14 respectively).

Group	***Animal I.D.***	***Gender (M/F)***	***Tapeworm FEC (e.p.g.)***	***Ascarid FEC (e.p.g.)***	***Mean consecutive strongyle FEC (e.p.g.) (±S.E.) D0***	***FEC D2 (e.p.g.)***	***FEC D14 (e.p.g.)***
Chigh							
	C-high1	F	0	0	125 (±11)	0	0
	C-high2	M	0	0	289 (±97)	0	0
	C-high3	M	0	0	131 (±0)	165.5(±14.5)	0
	C-high4	M	0	0	181 (±27)	0	0
	C-high5	M	0	0	271 (±79)	0.5(±0.5)	0
	C-high6	M	0	0	126 (±1)	0	0
	C-high7	M	0	0	100 (±1)	7 (±2)	0
	C-high8	M	0	0	162.5 (±5.5)	0	0
	C-high9	F	0	0	317 (±4)	3.5(±2.5)	0
Clow							
	C-low1	F	0	0	7.5 (±6.5)	0	0
	C-low2	M	0	0	0.5 (±0.5)	0	0
	C-low3	F	0	0	2.5 (±0.5)	0	0
	C-low4	F	0	0	4 (±1)	0	0
	C-low5	M	0	0	10 (±1)	0	0
	C-low6	F	0	0	0.5 (±0.5)	0	0
	C-low7	M	0	0	9.5 (±1.5)	0	0
	C-low8	M	0	0	2.5 (±1.5)	0	0
	C-low9	F	0	0	1 (±1.5)	0	0
	C-low10	M	0	0	0	0	0
	C-low11	M	0	0	1.5 (±0.5)	0	0
	C-low12	M	0	0	4.5 (±2.5)	0	0
	C-low13	F	0	0	7.5 (±0.5)	1(±1)	0
	C-low14	F	0	0	0	0	0

Supplementary Table S2: | Levels of selected faecal metabolites do not differ between animals according to parasite burden. The results of one-way Analyses of Variance (ANOVA) comparisons of faecal metabolite levels between samples from C-high (≥100 eggs per gram (e.p.g.)) and C-low (≤10 e.p.g.) at day 0 (pre-treatment). (P = p-value; FDR = False Discovery Rate; F = F value)

Metabolite	P	FDR	F	mean C-high	Mean C-low	Fold Change
Glutamate	0.053	0.28	4.2	1.71	1.18	1.45
Lysine	0.054	0.28	4.2	0.75	0.48	1.55
Glucose	0.054	0.28	4.2	1.66	1.01	1.65
Isobutyrate	0.061	0.28	3.9	3.05	2.59	1.18
Trehalose	0.062	0.28	3.9	0.23	0.099	2.29
Leucine	0.065	0.28	3.8	0.46	0.32	1.45
Phenylalanine	0.069	0.28	3.7	0.21	0.13	1.56
Propionate	0.084	0.29	3.3	18.81	17.43	1.08
betaAla	0.12	0.32	2.6	0.42	0.2	2.16
Valerate	0.13	0.32	2.5	2.85	3.31	-1.2
X3phenylpropionate	0.16	0.32	2.1	0.91	0.79	1.15
Nicotinate	0.16	0.32	2.1	0.072	0.091	-1.3
Valine	0.18	0.32	1.9	0.39	0.27	1.43
Tyrosine	0.18	0.32	1.9	0.24	0.18	1.38
Glycine	0.19	0.32	1.8	0.86	0.45	1.89
Alanine	0.19	0.32	1.8	1.15	0.76	1.53
Formate	0.2	0.32	1.7	0.07	0.058	1.21
Succinate	0.21	0.32	1.7	0.2	0.16	1.26
Butyrate	0.22	0.32	1.6	11.84	13.06	-1.1
Methionine	0.24	0.32	1.5	0.33	0.28	1.21
Isoleucine	0.24	0.32	1.5	0.4	0.31	1.32
Uracil	0.29	0.37	1.2	0.15	0.12	1.27
Inosine	0.53	0.65	0.4	0.08	0.071	1.13
Acetate	0.62	0.71	0.26	68.94	67.79	1.02
Asparagine	0.63	0.71	0.24	0.95	0.85	1.12
Aspartate	0.82	0.87	0.054	0.95	0.92	1.04
Phenylacetate	0.85	0.87	0.036	1.41	1.38	1.02
Isovalerate	0.87	0.87	0.029	2.21	2.17	1.02

Supplementary Table S3 | Levels of selected faecal metabolites in animals with high parasite infection burden do not differ before and after treatment with ivermectin. The results of one-way Analyses of Variance (ANOVA) comparisons of faecal metabolite levels of samples from C-high (≥100 eggs per gram (e.p.g.)) between day 0 (D0) (pre-treatment) and day 14 (D14) (post-treatment). (P = p-value; FDR = False Discovery Rate; F = F value).

Metabolites	P	FDR	F	mean D0	mean D14	Fold Change
Butyrate	0.0052	0.15	10	11.84	9.54	-1.2
Propionate	0.052	0.49	4.4	18.81	16.85	-1.1
Aspartate	0.081	0.49	3.5	0.95	1.29	1.35
Inosine	0.091	0.49	3.2	0.08	0.12	1.5
Uracil	0.11	0.49	2.9	0.15	0.21	1.38
Leucine	0.13	0.49	2.5	0.46	0.71	1.54
Isobutyrate	0.14	0.49	2.4	3.05	2.72	-1.1
Asparagine	0.14	0.49	2.4	0.95	1.11	1.17
Glucose	0.16	0.49	2.2	1.66	2.34	1.41
Phenylalanine	0.18	0.49	1.9	0.21	0.31	1.48
Isovalerate	0.2	0.49	1.8	2.21	1.88	-1.2
Tyrosine	0.21	0.49	1.7	0.24	0.35	1.44
Valine	0.24	0.52	1.5	0.39	0.58	1.5
Methionine	0.28	0.56	1.2	0.33	0.39	1.16
Isoleucine	0.36	0.67	0.9	0.4	0.54	1.33
Valerate	0.41	0.72	0.73	2.85	2.59	-1.1
betaAla	0.45	0.74	0.6	0.42	0.29	-1.5
X3phenylpropionate	0.6	0.93	0.29	0.91	0.86	-1.1
Phenylacetate	0.69	0.94	0.16	1.41	1.36	-1
Trehalose	0.76	0.94	0.097	0.23	0.25	1.11
Succinate	0.77	0.94	0.089	0.2	0.21	1.04
Glutamate	0.79	0.94	0.077	1.71	1.63	-1.1
Lysine	0.86	0.94	0.033	0.75	0.78	1.04
Glycine	0.86	0.94	0.033	0.86	0.79	-1.1
Acetate	0.88	0.94	0.024	68.94	68.53	-1
Formate	0.91	0.94	0.012	0.07	0.068	-1
Nicotinate	0.93	0.94	0.0076	0.072	0.071	-1
Alanine	0.94	0.94	0.0067	1.15	1.19	1.03

Supplementary Table S4: | Levels of selected faecal metabolites in animals with low parasite infection burden differ before and after treatment with ivermectin. The results of one-way Analyses of Variance (ANOVA) comparisons of faecal metabolite levels in samples from C-low (≤10 eggs per gram (e.p.g.)) between day 0 (D0) (pre-treatment) and day14 (D14) (post-treatment). (P = p-value; FDR = False Discovery Rate; F = F value).

Metabolites	P	FDR	F	mean D0	mean D14	Fold Change
Glucose	8.2E-07	2.3E-05	41	1.01	2.22	2.21
Uracil	1.8E-05	0.00025	28	0.12	0.27	2.29
Inosine	0.00011	0.001	21	0.071	0.14	2.02
Trehalose	0.00041	0.0029	16	0.099	0.21	2.12
Leucine	0.004	0.022	10	0.32	0.58	1.84
Butyrate	0.0087	0.041	8.1	13.06	10.88	-1.2
Valine	0.011	0.044	7.5	0.27	0.47	1.73
Lysine	0.029	0.1	5.4	0.48	0.68	1.4
Alanine	0.039	0.12	4.7	0.76	1.02	1.36
Phenylalanine	0.047	0.12	4.3	0.13	0.23	1.71
Isoleucine	0.047	0.12	4.3	0.31	0.44	1.45
Tyrosine	0.05	0.12	4.2	0.18	0.27	1.52
Methionine	0.075	0.16	3.4	0.28	0.36	1.32
Glutamate	0.12	0.24	2.5	1.18	1.48	1.25
Aspartate	0.16	0.3	2	0.92	1.13	1.23
Glycine	0.18	0.32	1.9	0.45	0.57	1.26
Isovalerate	0.27	0.44	1.3	2.17	1.96	-1.1
Acetate	0.29	0.45	1.2	67.79	69.49	1.02
Valerate	0.32	0.47	1	3.31	3.09	-1.1
Succinate	0.37	0.51	0.85	0.16	0.18	1.15
Asparagine	0.38	0.51	0.79	0.85	1.02	1.19
Formate	0.48	0.58	0.51	0.058	0.066	1.14
betaAla	0.48	0.58	0.5	0.2	0.22	1.14
X3phenylpropionate	0.56	0.65	0.36	0.79	0.81	1.04
Nicotinate	0.59	0.66	0.29	0.091	0.085	-1.1
Propionate	0.61	0.66	0.27	17.43	17.01	-1
Phenylacetate	0.83	0.86	0.047	1.38	1.36	-1
Isobutyrate	0.88	0.88	0.025	2.59	2.62	1.01
